# Toxic Mechanisms of Five Heavy Metals: Mercury, Lead, Chromium, Cadmium, and Arsenic

**DOI:** 10.3389/fphar.2021.643972

**Published:** 2021-04-13

**Authors:** Mahdi Balali-Mood, Kobra Naseri, Zoya Tahergorabi, Mohammad Reza Khazdair, Mahmood Sadeghi

**Affiliations:** ^1^ Medical Toxicology and Drug Abuse Research Center, Birjand University of Medical Sciences, Birjand, Iran; ^2^ Cardiovascular Disease Research Center, Birjand University of Medical Sciences, Birjand, Iran

**Keywords:** heavy metals, mechanistic action, acute poisoning, chronic poisoning, ROS, oxidative stress

## Abstract

The industrial activities of the last century have caused massive increases in human exposure to heavy metals. Mercury, lead, chromium, cadmium, and arsenic have been the most common heavy metals that induced human poisonings. Here, we reviewed the mechanistic action of these heavy metals according to the available animal and human studies. Acute or chronic poisonings may occur following exposure through water, air, and food. Bioaccumulation of these heavy metals leads to a diversity of toxic effects on a variety of body tissues and organs. Heavy metals disrupt cellular events including growth, proliferation, differentiation, damage-repairing processes, and apoptosis. Comparison of the mechanisms of action reveals similar pathways for these metals to induce toxicity including ROS generation, weakening of the antioxidant defense, enzyme inactivation, and oxidative stress. On the other hand, some of them have selective binding to specific macromolecules. The interaction of lead with aminolevulinic acid dehydratase and ferrochelatase is within this context. Reactions of other heavy metals with certain proteins were discussed as well. Some toxic metals including chromium, cadmium, and arsenic cause genomic instability. Defects in DNA repair following the induction of oxidative stress and DNA damage by the three metals have been considered as the cause of their carcinogenicity. Even with the current knowledge of hazards of heavy metals, the incidence of poisoning remains considerable and requires preventive and effective treatment. The application of chelation therapy for the management of metal poisoning could be another aspect of heavy metals to be reviewed in the future.

## Introduction

Heavy metals have harmful effects on human health, and exposure to these metals has been increased by industrial and anthropogenic activities and modern industrialization. Contamination of water and air by toxic metals is an environmental concern and hundreds of millions of people are being affected around the world. Food contamination with heavy metals is another concern for human and animal health. Concentration of heavy metals in water resources, air, and food is assessed with this regard (Mousavi et al., 2013; Ghorani-Azam et al., 2016; Luo et al., 2020). Metals among the other environmental pollutants may also occur naturally and remain in the environment. Hence, human exposure to metals is inevitable, and some studies have reported gender differences in the toxicity of metals (Vahter et al., 2007; Tchounwou et al., 2012). They may frequently react with biological systems by losing one or more electrons and forming metal cations which have affinity to the nucleophilic sites of vital macromolecules. Several acute and chronic toxic effects of heavy metals affect different body organs. Gastrointestinal and kidney dysfunction, nervous system disorders, skin lesions, vascular damage, immune system dysfunction, birth defects, and cancer are examples of the complications of heavy metals toxic effects. Simultaneous exposure to two or more metals may have cumulative effects (Fernandes Azevedo et al., 2012; Cobbina et al., 2015; Costa, 2019; Gazwi et al., 2020). High-dose heavy metals exposure, particularly mercury and lead, may induce severe complications such as abdominal colic pain, bloody diarrhea, and kidney failure (Bernhoft, 2012; Tsai et al., 2017). On the other hand, low-dose exposure is a subtle and hidden threat, unless repeated regularly, which may then be diagnosed by its complications, e.g., neuropsychiatric disorders including fatigue, anxiety, and detrimental impacts on intelligence quotient (IQ) and intellectual function in children (Mazumdar et al., 2011). The fact that several metals have emerged as human carcinogens is another important aspect of the chronic exposure. While the exact mechanism is unclear, aberrant changes in genome and gene expression are suggested as an underlying process. Carcinogenic metals such as arsenic, cadmium, and chromium can disrupt DNA synthesis and repair (Clancy et al., 2012; Koedrith et al., 2013). The toxicity and carcinogenicity of heavy metals are dose dependent. High-dose exposure leads to sever responses in animal and human which causes more DNA damage and neuropsychiatric disorders (Gorini et al., 2014). The toxic mechanism of heavy metals functions in similar pathways usually via reactive oxygen species (ROS) generation, enzyme inactivation, and suppression of the antioxidant defense. However, some of them cause toxicities in a particular pattern and bind selectively to specific macromolecules. Different toxic mechanisms of heavy metals increase our knowledge on their harmful effects on the body organs, leading to better management of animal and human poisonings. We aimed to review the literature on the toxicity mechanisms associated with heavy metals, which will increase our knowledge on their toxic effects on the body organs, leading to better management of the metal poisonings.

## Methods

This review provides context and specific overview of the currently available research data on the toxic mechanisms of heavy metals. It was aimed at reviewing the toxic effects and mechanisms of five main heavy metals including mercury, lead, cadmium, chromium, and arsenic. Current study intended to discuss and compare the data on toxic mechanisms from main scientific databases including PubMed, Web of Science (ISI), Scopus, and Google Scholar. The search terms included “mercury,” “lead,” “cadmium,” “chromium,” “arsenic,” “toxicity,” “poisoning,” “intoxication,” “mechanism of toxicity,” “mechanism of action,” and “cancer”. The literature was searched for animal and human studies involving acute and chronic exposures to the five metals and any related harmful adverse effects to body organs. Letter to the editor and unpublished data were excluded. Toxic mechanisms for each metal were extracted from the literature. Their similarities and differences were compared (Table 1).

**TABLE 1:** Toxic mechanisms of Hg, Pb, Cr, Cd, and As.

Toxic metal	Organ toxicity	Disrupted macromolecule/mechanism of action	References
Mercury (Hg)	- CNS injuries	- Thiol binding (GSH conjugation)	Cheng et al., 2006; Bottino et al., 2016; Chen R. et al., 2019; Zhang et al., 2020
	- Renal dysfunction	- Enzymes inhibition	
	- GI ulceration	- ROS production	
	- Hepatotoxicity	- Aquaporins mRNA reduction	
		- Glutathione peroxidase inhibition	
		- Increased c-fos expression	
Lead (Pb)	- CNS injury	- Increased inflammatory cytokines IL-1β, TNF-α, and IL-6 in the CNS	Struzynska et al., 2007; Dongre et al., 2011; Wang et al., 2013; Boskabady et al., 2016
	- Lungs dysfunction	- Increased serum ET-1, NO, and EPO	
	- Hematological changes (Anemia)	- Inactivation of δ-ALAD and ferrochelatase (inhibition of heme biosynthesis)	
	- GI colic	- Reduced GSH, SOD, CAT, and GPx levels	
	- Liver damage		
	- Reduced pulmonary function		
	- Cardiovascular dysfunction		
Chromium (Cr)	- Kidney dysfunction	- DNA damage	Deng et al., 2019; Pavesi and Moreira, 2020
	- GI disorders	- Genomic instability	
	- Dermal diseases	- Oxidative stress and ROS generation	
	- Increasing the incidence of cancers including lungs, larynx, bladder, kidneys, testicular, bone, and thyroid		
Cadmium (Cd)	- Degenerative bone disease	- miRNA expression dysregulation	Schutte et al., 2008; Pan et al., 2013; Pi et al., 2015; Fay et al., 2018; Wang Y. et al., 2018
	- Kidney dysfunction	- Apoptosis	
	- Liver damage	- Endoplasmic reticulum stress	
	- GI disorders	- Cd-MT absorption by the kidneys	
	- Lungs injuries	- Dysregulation of Ca, Zn, and Fe homeostasis	
	- Disorders in the metabolism of Zn and Cu	- Low serum PTH	
	- Cancer	- ROS generation	
		- Altered phosphorylation cascades	
Arsenic (As)	- Cardiovascular dysfunction	-Damage of capillary endothelium	Jolliffe et al., 1991; Luo et al., 2012; Shen et al., 2013
	- Skin and hair changes	- Thiol binding (GSH conjugation)	
	- CNS injury	- Uncoupler of oxidative phosphorylation (inhibition of ATP formation)	
	- GI discomfort	- Alterations in neurotransmitter homeostasis	
	- Liver damage		

## Toxic Effects of Heavy Metals

### Mercury (Hg)

Mercury (Hg) is found in air, water, and soil and exists in three forms: elemental or metallic mercury (Hg^0^), inorganic mercury (Hg^+^, Hg^2+^), and organic mercury (commonly methyl or ethyl mercury) (Li R. et al., 2017). Elemental mercury is liquid at room temperature and can be readily evaporated to produce vapor. Mercury vapor is more hazardous than the liquid form. Container breakage causes Hg^0^ spills and inhaling large amounts of Hg vapor can be fatal. Organic mercury compounds such as methyl mercury (Me-Hg) or ethyl mercury (Et-Hg) are more toxic than the inorganic compounds. The order of increasing toxicity related to different forms of mercury is defined as Hg^0^ < Hg^2+^, Hg^+^ < CH_3_-Hg (Kungolos et al., 1999). Mercury compounds have many applications in mining for example extraction of gold and some industrial processes. In lamp producing factories, Hg is used in the production of fluorescent light bulbs. Me-Hg and Et-Hg have been used as fungicides to protect plants against infections. Moreover, mercury has had medicinal uses in the past, but such drugs have been replaced by safer pharmaceutical medicines. Some examples are chlormerodrin, merbaphen, and mercurophylline (all diuretics) and phenylmercury nitrate (disinfectant). Besides, some skin lightening creams and some soaps are mercury polluted. Mercury chloride (HgCl_2_) is one of the active ingredients of skin brightening creams which are used to remove freckles and spots of the skin due to excessive accumulation of melanin. HgCl_2_ inhibits tyrosinase activity irreversibly, an enzyme which functions in melanin formation, by replacing the copper cofactor (Chen et al., 2020). Further, a mercury-containing organic compound called thimerosal has been used as a preservative in multidose vials of vaccines. Hg^0^ (vapor) is readily absorbed from lungs (80%) and distributed throughout the body. Hg^0^ can pass the blood brain barrier (BBB) and placenta; thus, its neurotoxicity is higher than inorganic Hg which passes through membranes at a slower rate. Hg^0^ is oxidized in the body to produce divalent Hg (Hg^2+^). Hg^0^ (liquid) is slightly absorbed from the gastrointestinal (GI) tract and does not appear to be toxic. Inorganic Hg is concentrated in the kidneys—reabsorbed from proximal tubules as Cys-S-Hg-S-Cys or basolateral membrane by organic anions transporters. Inorganic Hg cannot pass the BBB and placenta. Organic Hg is easily absorbed from the GI tract (95%) and distributed throughout the body. CH_3_-Hg is bound to thiol-containing molecules such as cysteine (CH_3_-Hg-Cys) so that it can pass the BBB. Hair is considered as an index of Hg exposure since CH_3_-Hg is accumulated there. Other than hair, Hg is excreted in urine and feces. CH_3_CH_2_-Hg follows similar pharmacokinetics to CH_3_-Hg (Bridges and Zalups, 2017).

#### Animal Studies

In an animal model of acute toxicity, exposure to Hg vapor (550 μg/m^3^) resulted in mercury deposits in different parts of the brain and also in the spinal cord (Møller-Madsen, 1992). Vapor exposure can cause a 70% increase in brain metallothionein, a metal binding protein, in the mice (Yasutake et al., 2004). Aragão et al. (2018) demonstrated cognitive impairment and hippocampal damage in rats with oral chronic administration of HgCl_2_. They also found that mercury levels in the hippocampus increased to 0.04 μg/g while the control group had Hg concentrations less than 0.01 μg/g (Aragão et al., 2018). Another study indicated methylmercury chloride induced CNS injury in rats receiving different doses of 0.05, 0.5, and 5 mg/kg Hg against normal saline group. CNS damage was showed via the increased expression of c-fos protein in cortex and hippocampus as an important signal transduction pathway. Hg accumulation in the brain was also observed in treated rats (Cheng et al., 2006). Chronic mercury vapor exposure can cause renal injury. Investigation of kidney tissues of Wistar rats exposed to 1 mg/m^3^ Hg vapor per day revealed histological alteration of the kidneys after 45 days (Akgül et al., 2016). However, the renal Hg deposition might be slow by the inhibition of γ-glutamyltranspeptidase, resulting in increased urinary Hg concentration. The basis for such reduction refers to the role of γ-glutamyltranspeptidase in the reuptake of mercury-glutathione conjugate in glomerulus (Kim et al., 1995). Following renal injury related to HgCl_2_, the antioxidative system of the rat kidney is compromised. Decreasing glutathione peroxidase activity and increased contents of oxidative lipids and proteins occurred as well as morphological changes in renal tissue 72 h after intoxication (Velyka et al., 2014). Only 4 days of oral exposure to Hg compounds (HgCl_2_ and Me-HgCl) in rats caused intestinal fluid accumulation, mucosal injuries, and diarrhea. Administration of Hg (5 mg/kg) reduced mRNA and downregulated aquaporins in the rat gastrointestinal tract. Impaired aquaporins expression seems to be involved in the GI tract harmful effects related to Hg intake (Bottino et al., 2016). Hg-induced hepatotoxicity was shown in an HgCl_2_ treatment animal model. The hepatotoxicity of mercury was determined by histopathological analysis and investigating elevated liver enzymes level including alanine transaminase (ALT), aspartate transaminase (AST), and alkaline phosphatase (ALP). An Hg-induced hepatotoxicity study showed gender differences in Wistar rats and female rats had higher levels of Hg accumulation in the liver. It was suggested that sex-dependent effects of liver damage via Hg exposure be related to the organic anion transporter 3 (Oat3). Male rats showed decreased expression of Oat3. The decrease in the Oat3 abundance will limit Hg intake into the liver, leading to lower hepatotoxicity in males (Hazelhoff and Torres, 2018). It seems that the organic anion transport is regulated by sex hormones. Ljubojevic et al. (2004) showed that the expression of Oat3 protein is strongly elevated by testosterone. The expression of these transporters may be regulated by sex hormones. Hormone-dependent expression of Oat3 transporter might be responsible for these differences (Ljubojevic et al., 2004). Bottino et al. (2016) proposed that activation of the Nrf2 signaling pathway could be effective to prevent liver lesions induced by Hg. ROS production reduces in this way and thus oxidative stress decreases (Bottino et al., 2016).

#### Human Studies

Microorganisms in marine environments perform natural biomethylation reactions to produce Me-Hg. Me-Hg enters the food chain of aquatic animals and eventually enters the human body through the consumption of fish (Moriarity et al., 2020). Cooking fish does not diminish its Hg content (Perello et al., 2008). An incident of exposure to organic Hg via the consumption of contaminated fish occurred in Minamata Bay, Japan, in the middle of 1950s. Soon afterward, the illness came to be known as Minamata disease. Chronic Hg toxicity caused neurological damage including ataxia, muscle weakness, numb limbs, disturbance in speech, chewing, and swallowing, and brisk and increased tendon reflex among the patients exposed to massive amounts of Me-Hg. Infants with severe developmental disabilities were born from the poisoned pregnant women (Dos Santos et al., 2018). Lethal Hg intoxication is usually due to the accidental exposure. Findings of neural electromyography among 104 people who had occupational exposure to mercury, with median urinary Hg concentration of 88.5 μg/g creatinine, manifested chronic injuries to peripheral nerves (Li L. et al., 2017). Hg preferentially accumulates in the kidneys and exerts deleterious effects especially in the proximal tubules. Chen R. et al. (2019) investigated the effects of Hg exposure on nonalcoholic fatty liver disease in 6,389 adolescents aged 12–17 years. The median blood Hg level was 0.73 ± 0.91 μg/L and a mild ALT elevation was observed (Chen R. et al., 2019). The relationship between blood Hg concentrations and the level of liver enzymes including ALT, AST, and gamma glutamyl transferase (GGT) was significant especially in elderly population (>60°years). Moreover, there is a potentiation effect with regular alcohol drinking and high blood Hg concentration (Lee et al., 2017). This might be explained by oxidative stress, impairment of cellular metabolism, and cell death due to liver damage via Hg exposure, leading to an elevation in the liver enzymes, especially in GGT level. In a cross-sectional study, the level of liver enzymes changed by the increase in blood Hg levels. The relationship between GGT and blood Hg concentration was significant, suggesting that GGT may be considered an early oxidative stress marker for mercury exposure (Choi et al., 2017). Two studies in China evaluated renal effects of Hg exposure among inhabitants near to mercury mines which were highly Hg polluted areas and found elevated levels of serum creatinine and urea nitrogen in comparison to those in the control areas. Hg-induced renal function impairment was revealed to be higher in females and the elderly people (Li et al., 2013; Li et al., 2015). Maternal exposure in such areas also could cause impaired renal function (Zhang et al., 2020).

### Lead (Pb)

Lead is a harmful environmental pollutant which has high toxic effects to many body organs. Even though Pb can be absorbed from the skin, it is mostly absorbed from respiratory and digestive systems. Pb exposure can induce neurological, respiratory, urinary, and cardiovascular disorders due to immune-modulation, oxidative, and inflammatory mechanisms. Furthermore, Pb could disturb the balance of the oxidant–antioxidant system and induce inflammatory responses in various organs. Exposure to Pb can produce alteration in physiological functions of the body and is associated with many diseases (Joseph et al., 2005; Jacobs et al., 2009; Kianoush et al., 2012). Pb is highly toxic which has adverse effects on the neurological, biological, and cognitive functions in the bodies. The international level-of-concern for Pb poisoning is 10 μg/dl in the blood (Burki, 2012; Kianoush et al., 2013). Adulteration of opium with Pb has been considered as a threat to human health in recent years (Kianoush et al., 2015).

#### Animal Studies

Daily administration of lead acetate (PbA) (15 mg/kg, ip) for 2 weeks in pregnant Wistar female rats induced proinflammatory cytokines including IL-1β and TNF-α in the hippocampus and IL-6 in the forebrain of immature rat brain. These results suggested that chronic lead exposure causes inflammation in the central nervous system (CNS) of immature rat brain which might be through activation of glial cells (Struzynska et al., 2007). Lead exposure (0.1–0.4 MPb) increased serum endothelin-1 (ET-1), serum nitric oxide (NO), and eosinophil peroxidase (EPO) accompanied with lung pathological changes in ovalbumin (OA) sensitized and nonsensitized guinea pigs (Boskabady et al., 2016). Exposure to PbA (0.1–0.4 MPb) increased serum total protein, histamine, and total and differential white blood cells (WBC) counts of guinea pigs (Farkhondeh et al., 2014). Hematological effects of chronic toxicity of the PbA (0.4%) in drinking water for 12 weeks in adult male rats showed significantly decreased red blood cells (RBC) count, while increasing total and differential WBC counts (Mugahi et al., 2003). Subchronic exposure to Pb at 10 and 100 times the normal concentrations via drinking water in rats increased activities of AST, ALP, and levels of urea nitrogen and creatinine. Besides, exposure to Pb induced necrotic changes in brain, liver, and kidneys (Jadhav et al., 2007). Intraperitoneal administration of PbA (10 mg/kg, bw) to induce hepatotoxicity in rats enhanced hepatocyte abnormalities and increased acid phosphatases (APs), ALP, AST, ALT, and lactate dehydrogenase (LDH) levels (Abirami et al., 2007). Exposure of rats to PbA significantly reduced antioxidant parameters such as glutathione peroxidase (GPx), catalase (CAT), superoxide dismutase (SOD), and glutathione S-transferase (GST) and decreased glutathione (GSH) content in hepatocytes and erythrocytes. In addition, Pb significantly increased oxidative parameters including malondialdehyde (MDA) and H_2_O_2_ concentrations as well as expression of cyclooxygenase-2 (COX-2) (Omobowale et al., 2014). Adult male rats exposed to PbA (500 mg/L) showed significant elevation in the levels of serum ALT, AST, urea, uric acid, and creatinine. PbA also increased lipid peroxide and reduced GSH, SOD, CAT, and GPx levels in liver and kidney tissues of induced animals (Wang et al., 2013). Administration of Pb (1 mg/kg, bw, ip.) in rats for a period of 4 weeks significantly increased lipid peroxide level in the liver, but antioxidant parameters such as SOD and CAT activities did not change statistically (Patra et al., 2001). Subcutaneous administration of PbA (100 mg/kg, bw) in rats increased the levels of lipid peroxidation (LPO) including MDA and 4-hydroxyalkenals (4-HDA) levels in the liver and kidneys compared to the control animals. Moreover, PbA decreased SOD and total GSH in the liver and kidneys of rats (El-Sokkary et al., 2005). Administration of PbA in rat diet (500 mg/kg/day) for 2, 4, and 6 weeks resulted in the elevations of serum ALT, AST, GGT, and ALP activities and hepatic necroinflammatory lesions. Lead also induced hepatocyte proliferation, portal inflammatory infiltrate, steatosis, apoptosis, and mild fibrosis 6 weeks after administration (Shalan et al., 2005). It has been reported that PbA (500 mgPb/L) in drinking water promoted apoptosis by activation of mitochondria cytochrome C release and inhibited Bcl-2 proteins and activation of caspase-3 in kidneys of the exposed rats (Liu et al., 2012). In a study, administration of PbA (25 mg/kg, bw, ip.) for 5 days in rats increased urea and creatinine levels while it decreased total protein and albumin in the serum. The reduction of antioxidant parameters including GPx, CAT, and SOD and induction of hyperlipidemia resulted from lead induced toxicity. Furthermore, hemorrhage in the renal tubules, degeneration in epithelial cells, disruption of Bowman’s capsule, and necrosis area in kidney tissue were observed (Abdou and Hassan, 2014). Orally administered PbA (30 and 60 mg/kg, bw) 3 days in a week for 3 months in rats reduced testicular weight, sperm count, and testicular ALP activity. PbA also induced abnormal sperm count, seminiferous tubules necrosis, and disappearance of seminiferous tubular epithelium in a dose-dependent manner (Sujatha et al., 2011).

#### Human Studies

**FIGURE 1:**
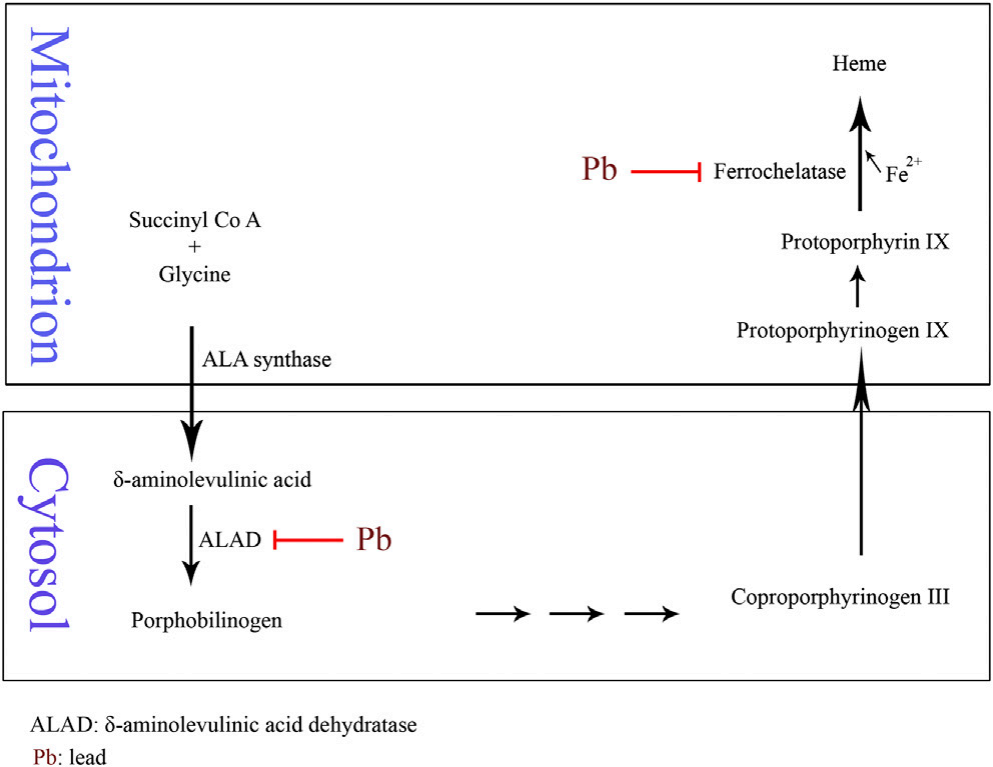
Schematic of Pb-induced anemia via the inhibition of δ-aminolevulinic acid dehydratase and ferrochelatase enzymes in heme biosynthesis.

Anemia may develop with Pb poisoning via the inhibition of ferrochelatase and δ-aminolevulinic acid dehydratase (ALAD), the two of many enzymes involved in heme biosynthesis (Figure 1). The inhibition of ferrochelatase and ALAD by lead decreases heme synthesis which leads to anemia (Mense and Zhang, 2006). Several antioxidant molecules such as GSH and GSSG as well as antioxidant enzymes including SOD, CAT, GPx, and glutathione reductase (GR) may have fluctuations due to Pb exposure and consequent oxidative stress. Pb has high affinity to the reactive –SH group of GSH and is able to decrease the GSH levels. GPx, CAT, and SOD are metalloproteins that their antioxidant functions to detoxifying free radicals could be affected due to Pb exposure. Lead can induce oxidative damage in different organs via direct effect on membrane lipid peroxidation and reducing antioxidant parameters (Gurer-Orhan et al., 2004; Kasperczyk et al., 2005). It has been reported that low level of lead exposure exerts immunostimulating effects in contrast to higher exposure that usually leads to immunosuppression (Mishra, 2009). The results of a study showed that lymphocyte proliferation to phytohemagglutinin (PHA) is inhibited in Pb exposed population compared to the unexposed subjects. However, there was no correlation between inhibition of lymphocyte proliferation and blood lead level (BLL). In addition, Pb exposure did not affect natural killer (NK) cell cytotoxicity compared to the control group. On the other hand, interferon-γ (IFN-γ) was significantly elevated in Pb exposed individuals. It was shown that there was a significant positive correlation between BLL and IFN-γ level (Mishra et al., 2003). The immunological effects of lead exposure on lymphocyte proliferation, IFN-γ production, and NK cell cytotoxicity in occupational Pb exposed subjects including wheeler drivers (30), battery workers (34), silver jewelry makers (20), and unexposed healthy subjects (30) were studied. The results showed that lymphocyte proliferation to PHA is inhibited while IFN-γ significantly increased in stimulated peripheral blood mononuclear cells (PBMCs) compared with control subjects (Mishra et al., 2003). The pulmonary function tests (PFT) and respiratory symptoms in 108 battery manufacturing workers and 100 control subjects were evaluated. The lead concentrations in serum and urine of workers were significantly higher while the PFT values were significantly lower than the control group. In addition, the frequencies of respiratory symptoms including chest tightness (26%), cough (17%), and sputum (16%) were significantly higher in battery manufacturing workers compared to the control group (Khazdair et al., 2012). The association between BLL, serum immunoglobulin E (IgE), eosinophil count, and prevalence of asthma in a cross-sectional study of 1,788 children in 2005–2006 was investigated. The results of this study indicated an association between IgE and eosinophilia with lead exposure (Wells et al., 2014). The association of BLL to risk of developing asthma was studied in 4,634 children (1–3 years) from 1995 through 1998. It was found that BLL ≥ 5 μg/dl was not associated with asthma among African Americans while there was a slight but not significant association among Caucasians (Joseph et al., 2005). The relationship between blood and urine lead concentrations and clinical manifestations in car battery manufacturing workers showed a weak correlation between serum ALP and BLL in these workers (Dadpour et al., 2016). Pb exposure on 30 automobile workers remarkably increased blood (364%) and urinary (176%) lead levels compared to the control subjects. Systolic blood pressure (SBP), diastolic blood pressure (DBP), and total white blood cell count (11.44%) were increased in the automobile workers as compared to the controls. Also, red blood cell count, hemoglobin (Hb), hematocrit (Hct), mean corpuscle volume (MCV), and mean corpuscle hemoglobin (MCH) were significantly decreased in workers compared to the control group (Dongre et al., 2011). Moreover, Schober et al. (2006) reported the association of chronic exposures to high occupational Pb with atherosclerosis and cardiovascular mortality (Schober et al., 2006).

### Chromium (Cr)

Chromium (Cr) is found in the earth’s crust and seawater and is a naturally occurring heavy metal in industrial processes (Tchounwou et al., 2012). Cr has multiple oxidation states ranging from −2 to + 6, in which the trivalent and hexavalent forms are the most common stable forms (Shekhawat et al., 2015). Cr (VI) is related to a series of diseases and pathologies while Cr (III) is required in trace amounts for natural lipid and protein metabolism and also as a cofactor for insulin action (Cefalu and Hu, 2004; Achmad et al., 2017; Vincent, 2017; Vincent, 2019). Based on the International Agency for Research on Cancer (IARC) report (2018), hexavalent chromium has been classified as a group I occupational carcinogen (Loomis et al., 2018). In this context, a meta-analysis of 973,697 workers involving 17 standardized incidence ratios (SIRs) from seven countries and four kinds of occupations found that 11,564 of them had cancer (Deng et al., 2019). The primary route of exposure for nonoccupational human populations occurs via ingestion of chromium containing food and water or dermal contact with products containing chromium (Nickens et al., 2010). Furthermore, metallurgical, refractory, and chemical industries release a large amount of Cr into soil, ground water, and air which causes health issues in humans, animals, and marine life (Fang et al., 2014). Cr can cause a variety of diseases through bioaccumulation in human body. This ranges from dermal, renal, neurological, and GI diseases to the development of several cancers including lungs, larynx, bladder, kidneys, testicles, bone, and thyroid (Fang et al., 2014).

#### Animal Studies

Wang et al. (2012) used a mouse model of colorectal cancer to investigate the carcinogenicity of Cr (VI) and As in drinking water. It was found that exposure to Cr and As increased the tumor incidence and size. It was mediated through the Wnt/beta-catenin signaling pathway induced by ROS production (Wang et al., 2012). Interactions of ROS and reactive nitrogen species (RNS) with cellular macromolecules such as DNA, lipid, and proteins are considered as one of the main leading causes of toxicity and cancer progress in Cr exposure (Aggarwal et al., 2019). In one study by Patlolla et al. (2009) on Sprague-Dawley rats, potassium dichromate was administered intraperitoneally for 5 days at the doses of 2.5, 5.0, 7.5, and 10 mg/kg body weight per day. A significant increase in the levels of ROS and malondialdehyde in liver and kidney along with a dose-dependent increase in superoxide dismutase and catalase activities was reported. Furthermore, after 24, 48, 72, and 96 h after treatment DNA damage was observed in a dose- and time-dependent manner (Patlolla et al., 2009). Salama et al. (2016) showed a high risk of brain damage in low doses of Cr on Albino Wistar rats via intranasal exposure. Moreover, higher Cr concentrations caused lung injury. A 2 mg/kg intranasal potassium dichromate resulted in the elevation of oxidative stress biomarkers: interleukin-1β (IL-1β), phosphorylated protein kinase B (PKB), and cyclooxygenase 2 (COX-2) (Salama et al., 2016).

#### Human Studies

The results of a recent meta-analysis showed exposure to Cr (VI) may cause increased mortality and incidence of some cancers including lung, larynx, bladder, kidney, testicular, bone, and thyroid cancer in humans (Deng et al., 2019). Moreover, an ecological study in 2011 in Greece found high levels of Cr (IV) in drinking water, 41–156 μg/L, and increased incidence of liver (*p* < 0.001), lung (*p* = 0.047), and genitourinary cancers (*p* = 0.025) among women (Linos et al., 2011). Another study in 2012 in India showed increased incidence of GI and dermatological complications among population exposed to Cr (VI) contaminated ground water (Sharma et al., 2012). DNA damage, genomic instability, and ROS generation are accepted as mechanisms of Cr toxicity and carcinogenicity. Both Cr (VI) and Cr (III) are capable of producing ROS (Pavesi and Moreira, 2020). Cr carcinogenicity is followed by DNA damage as disruption of transcription regulation. Furthermore, chromatin architecture is disrupted which causes shifts in chromatin accessibility and local and genome-wide nucleosomal position shifts (Schnekenburger et al., 2007). Cr-induced DNA damage includes DNA–Cr–protein crosslinks, DNA inter/intrastrand crosslinks, single- and double-strand breaks (SSBs and DSBs, respectively), and p53 point mutations (Urbano et al., 2012; Thompson et al., 2013). *In vitro* studies have demonstrated the ability of Cr (III) to bind nucleic acids (Urbano et al., 2012; Fang et al., 2014). Cr (III) forms binary and ternary DNA adducts. Ternary adducts are predominant and more toxicologically relevant in mammalian cells (Salnikow and Zhitkovich, 2008; Zhitkovich, 2011). Cr (III) ascorbate, Cr (III) cysteine, Cr (III) histidine, and Cr (III) glutathione DNA adducts are four types of ternary adducts (Salnikow and Zhitkovich, 2008). Cr (III) forms DNA–protein crosslinks in human lung adenocarcinoma cells (A549) (Macfie et al., 2010). SSBs and DSBs were found in rapidly dividing cells such as cancer cells as the collapse of replication fork during DNA replication, leading to cell cycle arrest and apoptosis via activation of p53 (Lee et al., 2016; Wilhelm et al., 2020). In one study by Ueno et al. SSBs were detected in the liver and kidney of male albino mice by a single injection of Cr (VI) but there were no significant levels of DNA strand breaks in the spleen, lung, or brain (Urbano et al., 2012). Hydroxyl radicals generated during the reduction of Cr (VI) by GSH can lead to hydrogen abstraction from the ribose moiety and strand break formation. In general, oxidative DNA damage occurs either by the oxidation states of Cr(V) peroxo intermediates or via ROS action (Bokare and Choi, 2011). SSBs, DSBs, and protein-Cr-DNA crosslinks were indicated in the liver cells of F344 rats orally exposed to potassium chromate via drinking water for 3 weeks (Coogan et al., 1991). Besides, *in vivo* and *in vitro* studies demonstrated DNA damage, microsatellite instability, and chromosome aberrations due to Cr (VI) exposure (Wise and Wise, 2012). It seems that factors including the tissue, cell type, Cr (VI) concentration, time of exposure (Ferreira et al., 2019), free radicals formation (DeLoughery et al., 2014), and reactivity of the intermediate Cr (V) and Cr (IV) (O’Brien et al., 2009) are involved in Cr-induced carcinogenicity. Cr (VI) itself does not bind to macromolecules or DNA instead; the hexavalent cation reacts with cellular reductants to produce Cr (V), Cr (IV), and Cr (III) (Zhitkovich, 2005). These metabolic intermediates generated during Cr (VI) reduction trigger Fenton-type reactions which generate hydroxyl radicals in the presence of hydrogen peroxide. Thus, oxidative stress is induced (Yao et al., 2008). Furthermore, formation of Cr-Asc (Ascorbate), Cr-GSH, and Cr-cys (cysteine) crosslinks depletes cellular antioxidants and induces oxidative stress (Yao et al., 2008). Cr (VI) induced oxidative stress and ROS production in high levels target DNA and lipid content of cells which leads to DNA damage and lipid peroxidation, respectively. Moreover, many other cellular injuries and cell death as both apoptosis and necrosis may occur. ROS production in medium to low levels in cells can lead to tumor formation and progression (De Flora et al., 2008). One study showed that the reduced-to-oxidized glutathione ratio (GSH/GSSG) is time and dose dependent (Thompson et al., 2011).

### Cadmium (Cd)

Cadmium (Cd), although rare, occurs naturally in soil and minerals such as sulfide, sulfate, carbonate, chloride, and hydroxide salts as well as in water. High levels of Cd in water, air, and soil can occur following industrial activities which could be a substantial human exposure to Cd. Moreover, the ingestion of contaminated food will cause major exposure to Cd. Cd exposure may also occur through smoking, which is capable of elevating blood and urine Cd concentrations. Presence of Cd in contaminated water could disturb the necessary mechanisms in the body, possibly resulting in short-term or long-term disorders (Jiang et al., 2015; Richter et al., 2017; Cao et al., 2018). Cd is classified by the International Agency for Research on Cancer (IARC) as carcinogenic to humans (Group 1) (Kim et al., 2020). Occupational exposure to Cd may occur in alloy, battery, and glass production and in electroplating industries. Due to the importance of the subject, Cd level in the air is routinely monitored in some countries (Cancer, 1993). Rice, grains, and sea food have been found to be polluted by Cd (Chunhabundit, 2016); nonetheless, after oral intake, a small portion of Cd is absorbed. Tragically, the outbreak of Itai-itai disease in Japan was due to the mass Cd contamination of food and water supplies. The patients suffered from painful degenerative bone disease, kidney failure, and the GI and lungs diseases (Nishijo et al., 2017). Unlike low GI absorption, Cd is more efficiently taken from the lungs via industrial dust. Acute or chronic inhalation of Cd in industrial areas might lead to renal tubular dysfunction and lung injuries. Cd blood concentration in smokers is almost twice higher than that of nonsmokers. Batáriová et al. (2006) found Cd blood levels of 0.4 against 1.3 μg/L for nonsmokers vs. smokers in adult population (Batáriová et al., 2006). This seems to be related to the nature of tobacco plants to accumulate relatively high Cd concentrations in tissues especially in the leaves (Proshad et al., 2020).

#### Animal Studies

Disorders in the metabolism of Zn and Cu were found in Borowska et al.’s (2017) study on rats that were exposed to 1 and 5 mg/kg Cd. It was shown that after a 10-month treatment of female Wistar rats by 1 mg/kg Cd, zinc absorption decreased, while under exposure to 5 mg/kg Cd, its absorption increased after 3 and 24 months. Moreover, after 24-month exposure to 1 mg/kg Cd, Zn concentration in the liver and kidneys increased while spleen concentration of Zn decreased. Following exposure to high dose of Cd (5 mg/kg), after 17 months, Zn concentration increased in spleen and kidneys while its liver concentration was unaffected. Furthermore, in a similar vein, chronic low level and moderate exposure to Cd influenced Cu concentrations in serum and internal organs. On the basis of this experiment, chronic exposure to Cd results in redistribution of essential bioelements which leads to the retention of Zn and Cu in the liver and kidneys and in contrast a decrease in their concentrations in the serum (Borowska et al., 2017). Cd is taken up into the GI tract through the function of very different transporters. With excessive Cd exposure, the intestinal accumulation increases, particularly in the duodenum. Ohta and Ohba (2020) examined the GI absorption of Cd by means of different metal transporters including metallothionein (MT), divalent metal transporter 1 (DMT1), zinc importing protein 14 (ZIP14), Cu-transporting P-type ATPase (ATP7A), and Transient Receptor Potential Vanilloid 6 (TRPV6) in three distinct segments of rats’ intestine. It was found that intestinal concentrations of Cu and Fe decreased in contrast to the increased Cd level. Moreover, the elevation in duodenal Cd exposure caused the increase in the expression of the MTI, MTII, DMT1, ZIP14, ATP7A, and TRPV6 genes. According to their findings, Cd binds to MT and other metal transporters in the small intestine and accumulates in the tissue. As a result, the concentration of both Cu and Fe decreases in the GI and accordingly their absorption into the bloodstream declines (Ohta and Ohba, 2020). Other studies suggest that Cd decreases the absorption of Zn, Ca, and Cu resulting in the low dietary intake of such essential elements. Fay et al. (2018) investigated the molecular mechanisms of Cd nephrotoxicity in rat’s renal cortex. Sprague-Dawley rats received subcutaneous injections of cadmium chloride compound (0.6 mg/kg) for 12 weeks. Afterward, renal cortex miRNA expression was determined. Biochemistry results showed that in contrary to significant increase in urine volume and also the excretion of protein in the urine, urinary creatinine was lower in Cd-treated group. Hence, proximal tubule dysfunction associated with Cd kidney injury causes the development of polyuria and proteinuria. Further, urine microglobulin was significantly higher compared to the control saline group. Molecular assays indicated that Cd dysregulates the miRNA expression; hence, the altered miRNA expression may play a role in Cd-induced nephrotoxicity. The expression of 44 miRNAs was significantly increased while 54 miRNAs were significantly decreased following Cd treatment (Fay et al., 2018). Cadmium is known to pose hepatotoxic effects (Park et al., 2013; Pi et al., 2015). Depending on the conditions of exposure, its acute toxicity may occur as necrosis or apoptosis. Incubation of rat hepatocytes with 1–5 μmol/L Cd for short exposure times (6-12 h) resulted in apoptosis following cytochrome C release into the cytosol (Pham et al., 2006). Endoplasmic reticulum stress and the subsequent cell apoptosis is another proposed path of Cd toxicity (Chen et al., 2015). Chronic Cd-induced renal damage mainly involves the damage of glomerulus, especially the proximal tubules. Accumulation of Cd in the epithelial cells of the proximal tubules results in impaired reabsorption function, leading to polyuria, glucosuria, and low molecular weight proteinuria. Cd-induced nephrotoxicity is mediated via oxidative stress, apoptosis, and necrosis. Cd enters the renal epithelial cells either as free or bound forms including Cd-MT, Cd-GSH, and Cd-Cys. Free form is taken up via metal transporters, while the bound forms are taken up via the receptor-mediated pathway. Apart from apoptosis and necrosis, there is evidence that other mechanisms are also involved in proximal tubular injury. Changes in cell–cell adhesion, modulation of cellular signaling pathways, and autophagic responses may occur prior to cell death. The disruption of cell adhesion and cellular signaling cascades, in turn, can trigger autophagic responses in the cells. In a mild injury, the autophagic response may be adequate to repair the damage. However, apoptosis or autophagic cell death can occur in a severe injury. Necrosis of the proximal tubule can occur after widespread cell damage. It is also noteworthy that regardless of Cd uptake mechanism, Cd can accumulate in epithelial cells of the proximal tubule. At high tissue levels of Cd, metallothionein and GSH are overwhelmed, and the cells undergo injury. By the onset of proximal tubule dysfunction apoptosis begins in low number of cells (5%). Many cells undergo Kim-1 upregulation and autophagy. One of the earliest toxic effects of Cd is the disruption of cadherin-mediated cell–cell adhesion. This may cause mild level of oxidative stress. Effect of Cd on cadherin-mediated cell–cell junctions might be due to the interaction with extracellular Ca-binding regions of the macromolecule (Choong et al., 2014; Yang and Shu, 2015; Messner et al., 2016).

#### Human Studies

It is understood that Cd produces dispositional tolerance via binding to metallothionein (MT) protein. Such MT induction protects the liver by limiting the distribution of Cd to sensitive cellular macromolecules. However, highly increased levels of cadmium result in unbound and free Cd; hence, the toxic effects occur. Lech and Sadlik (2017) presented cadmium levels in human tissues in a ten-year examination in nonpoisoned population. Autopsies were done on brain, stomach, small intestine, liver, kidneys, heart, and the lungs. The lowest level of Cd was in the brain (0.02 μg/g digested samples). High Cd concentrations were measured in kidneys (16.0 μg/g) and liver (1.5 μg/g), as Cd is mainly distributed into these two internal organs (Lech and Sadlik, 2017). Cd bound to MT is readily taken up by the kidneys. The complex is very toxic for the kidney and is mechanistically involved in the chronic nephrotoxicity of Cd. Cadmium is able to mimic the function and behavior of essential metals. For instance, similar to zinc, Cd binds to albumin in plasma. Consequently, dysregulation of calcium, zinc, and iron homeostasis occurs (Schaefer et al., 2020). Cd-induced liver injury may be associated with the disturbance of calcium (Ca) homeostasis. Disruption of Ca homeostasis induces mitochondrial fragmentation via increased levels of dynamin-related protein 1 (Drp 1) (Xu et al., 2013). Osteoporosis and bone fracture could be observed following Cd toxicity. In Chen et al. study (2019), comparing residents of two cadmium-polluted areas with a control region showed the association between osteoporosis and high level of Cd intake. Although both men and women showed similar trends, the changes were significant in female population (Chen X. et al., 2019). Cd osteotoxic effects might be due to the decreased levels of serum PTH at high cadmium body burden which in turn induces calcium release from bone tissues (Schutte et al., 2008). Lv et al. (2017) found an inverse association between osteoporosis and the amount of Cd in body (Lv et al., 2017). Cd plays a key role in cardiovascular diseases caused by smoking such as peripheral artery disease and coronary heart disease. Li et al. (2019) found association between Cd exposure and the pathway for cardiovascular diseases using mediation analysis in a population of 4,304 middle-aged men and women. They clarified that part of the smoking-induced cardiovascular diseases is mediated through cadmium (Li et al., 2019). Cd may give rise to the occurrence of the kidney, lungs, pancreas, breast, prostate, and GI cancers (Lin et al., 2018; Djordjevic et al., 2019). A proposed mechanism highlights a sequence of incidents such as ROS generation, elevation of TNF-α level, Nrf2 upregulation, and ultimately aberrant gene expression, deregulation of cell proliferation, and apoptosis resistance (Wang Y. et al., 2018). Nephrocarcinogenicity of cadmium might happen after substantial exposure in chemical industries (Pesch et al., 2000). A hypothesis in this regard explains that the β-catenin signaling pathway plays a role in renal carcinogenesis, including Cd-induced nephrocarcinogenesis. Via this signaling, β-catenin protein which is a transcriptional factor is accumulated in the cytoplasm. Overexpression of β-catenin leads to evasion of ROS-dependent/β-catenin signaling which in turn contributes to carcinogenesis of kidney (Thevenod and Chakraborty, 2010; Son et al., 2012). Urinary cadmium has been frequently used to assess Cd exposure and the related consequences. Some studies have reported the relationship between prevalence of renal dysfunction or deficit in lung function and measured urinary Cd levels. There might be a dose-response relation in this regard (Chen et al., 2006; Moitra et al., 2013). Beta 2-microglobulin in urine can be considered as a useful marker of tubular proteinuria particularly in Cd-induced toxicity assessments. It has a promising role since there is as low as 360 μg/L beta 2-microglobulin in urine (Argyropoulos et al., 2017).

### Arsenic (As)

Arsenic as a harmful heavy metal is one of the main risk factors for the public health. Sources of As exposure are occupational or via the contaminated food and water. As has a long history of use, either as a metalloid substance or as a medicinal product. It is notoriously known as the king of poisons and poison of kings (Gupta et al., 2017). As is present as a contaminant in food, water, and environment. Arsenic exists in the forms of metalloid (As^0^), inorganic (As^3+^ and As^5+^), organic, and arsine (AsH_3_). The order of increasing toxicity of As compounds is defined as organic arsenicals < As^0^ < inorganic species (As^5+^ < As^3+^) < arsine (Shah et al., 2010; Sattar et al., 2016; Kuivenhoven and Mason, 2019). Primary As absorption is from the small intestine. Other routes of exposure are from the skin contact and by inhalation. Following distribution to many tissues and organs in the body including the lungs, heart, kidneys, liver, muscles, and neural tissue, As is metabolized to monomethylarsonic acid (MMA) and dimethylarsinic acid (DMA) in which the latter is the predominant form in the urinary excretion of As (Del Razo et al., 1997; Ratnaike, 2003). Acute and chronic As toxicity is related to the dysfunctions of numerous vital enzymes. Similar to the other heavy metals, As can inhibit sulfhydryl group containing enzymes which leads to their dysfunction. Moreover, As inhibits the pyruvate dehydrogenase by binding to the lipoic acid moiety of the enzyme. Pyruvate dehydrogenase inactivation can block the Krebs cycle and inhibits oxidative phosphorylation. As a result, ATP production decreases, resulting in cell damage (Shen et al., 2013). Furthermore, the damage of capillary endothelium by As increases vascular permeability, leading to vasodilation and circulatory collapse (Jolliffe et al., 1991).

#### Animal Studies

Oxidative stress and mitochondrial dysfunction may cause neurodegeneration. Two As species, sodium arsenite and DMA, induced apoptosis in cultured neurons of rat brain via activation of p38 and JNK3 MAP kinases, two enzymes that are activated by stress signals and mediate neuron apoptosis. Inorganic As form was more toxic and reduced neuron viability more. Besides, arsenite selectively activated p38 and JNK3 in cerebellar neurons (Namgung and Xia, 2001). In a rat hippocampus model, As neurotoxicity was attributed to the altered expression of N-methyl-d-aspartate (NMDA) receptor and changes of molecular expression of postsynaptic signaling proteins. A decline in the expression of NR2A subunit of NMDA receptor and variation in proteins of postsynaptic density protein 95 (PSD-95) and calcium/calmodulin-dependent protein kinase II (p-CaMKII) were observed (Luo et al., 2012). ROS generation and cytochrome C release can play a role in the hepatotoxic effects of As exposure. Study of male Wistar rats exposed to As showed oxidative stress and activation of caspase-9, an initiator of the apoptosis signaling. The accumulation of ROS is responsible for lipid bilayer damage which causes mitochondrial swelling (Bodaghi-Namileh et al., 2018). Biochemical analysis of serum of Sprague-Dawley rats showed that As^3+^ significantly decreased the activity of serum cholinesterase (ChE) in a dose-dependent manner. ChE is an important enzyme for the proper functioning of the nervous system. The decreased ChE activity may be associated with peripheral or CNS injury (Patlolla and Tchounwou, 2005).

#### Human Studies

Proinflammatory cytokines TNF-α, IL-6, IL-8, and IL-12 were elevated in the plasma of rural women exposed to low level arsenic. The level of anti-inflammatory cytokine IL-10 was decreased in the exposed population. DNA damage of airway cells was observed following As exposure and the level of DNA adduct 8-hydroxy-2’-deoxyguanosine (8OHdG) was elevated in comparison to the control group (Dutta et al., 2015). Human studies on the association between As-contaminated drinking water and adverse pregnancy outcomes have shown that As can easily cross the placenta, particularly during early gestation, leading to spontaneous abortion, stillbirth, preterm birth, and low birth weight (Hopenhayn et al., 2003; Rahman et al., 2010). The male reproductive system might be impaired through the NF-kB signaling pathway by AS^3+^. NF-kB cell signaling plays role in spermatogenesis and testis functioning (Chen et al., 2012). Arsenic tends to accumulate in the skin. Arsenical keratosis and hyperpigmentation of the skin might occur following As exposure. A cutaneous toxicity study using cultured human keratinocytes showed β1-integrin distribution. Decreased expression in keratinocytes by integrins could result in apoptosis aberration and cutaneous manifestation (Lee et al., 2006). Severe cases of intoxication may also develop skin cancer. Oxidative stress, chromosome anomalies, and altered growth factors expression are possible modes of action in As-induced carcinogenesis (Yu et al., 2006). As-exposed populations may experience liver lesions. Liver cancers hepatocellular carcinoma and angiosarcoma have been associated with As poisoning (Lu et al., 2001; Liaw et al., 2008). The axon of peripheral nerves continues to degenerate in As neurotoxicity. It is believed that a main cause of As neurological disorders is the ability to cause oxidative stress and mitochondrial dysfunction. Lipid peroxidation induced by oxidative stress leads to DNA damage and ultimately brain cell death (Mochizuki, 2019).

## Overview of Heavy Metals Toxicity

### Exposure to Heavy Metals and Environmental Pollution

**FIGURE 2:**
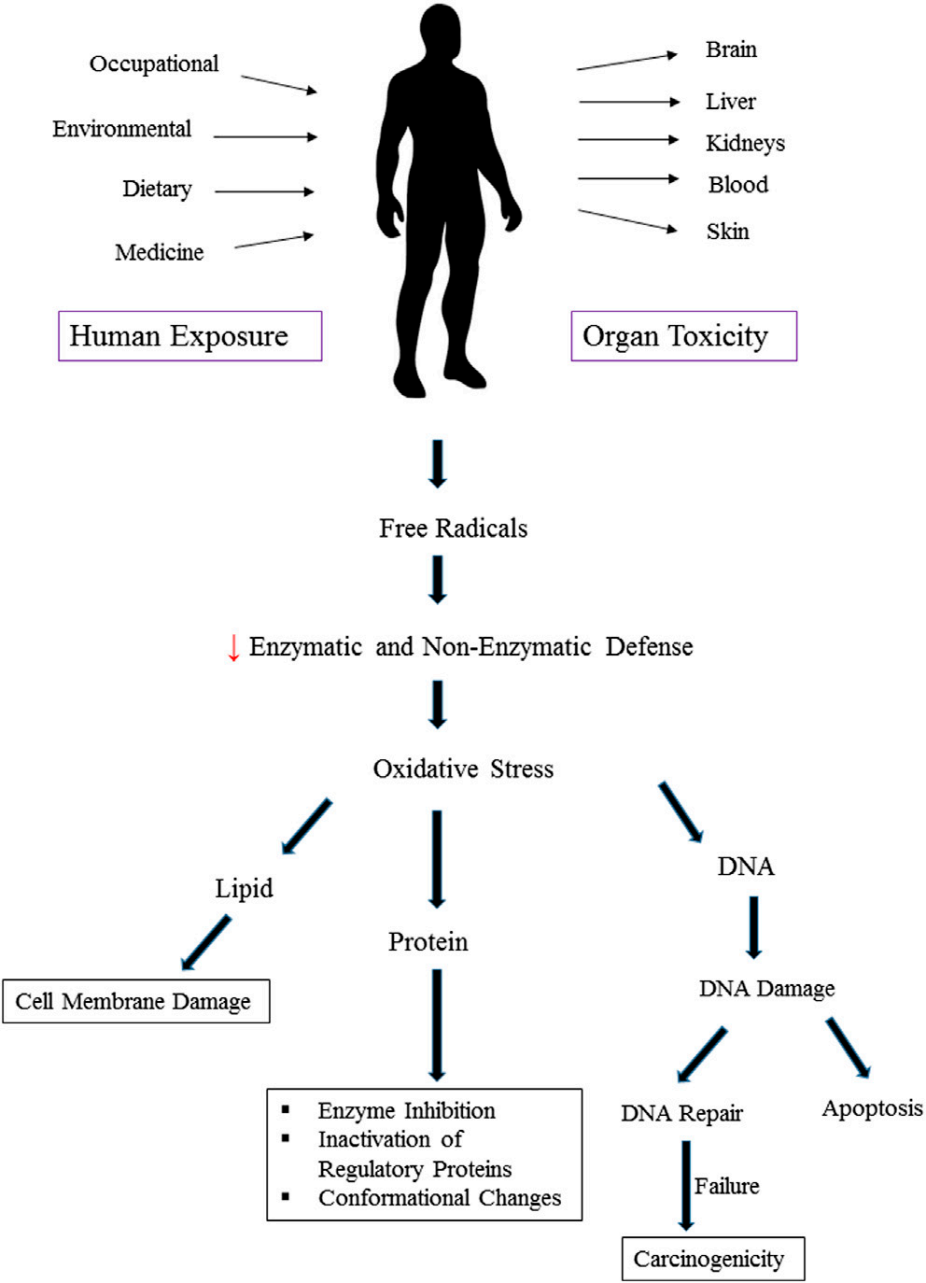
Oxidative stress and organ toxicity following exposure to heavy metals.

Heavy metals induce toxicity to biological systems via bonding to sulfhydryl groups and ROS generation (Figure 2). This causes inactivation of vital macromolecules and the occurrence of oxidative stress and depletion of glutathione. Following exposure to toxic metals and entrance to the body, various processes happen including interaction or inhibition of some metabolic pathways (Wu et al., 2016). As a result, numerous harmful effects on humans and animals are observed. These include specific organ dysfunctions, metabolic abnormalities, altered hormones, congenital disorder, immune system dysfunction, and cancer (Khazdair et al., 2012; Fang et al., 2014; Li L. et al., 2017; Deng et al., 2019). Therefore, many international organizations set standards regarding the presence of metals in the environment, foods, and drinking water. In risk assessment studies, the presence of heavy metals in food and water is checked. Saper et al. (2008) analyzed 193 Ayurvedic medicinal products, famous Indian herbal medicines, and found that about one-fifth contained Hg, Pb, and As. It was found that nearly 21% contained detectable levels of Pb, Hg, and As. Noticeably, all metal-containing products exceeded one or more standards for acceptable daily metal intake. This could result in Pb and/or Hg ingestions 100 to 10,000 times greater than acceptable limits (Saper et al., 2008). Besides, the level of metals that can reach the body on a daily basis is regulated. The daily dosage limit for these heavy metals (μg/day) is presented in Table 2. Daily dosage limits for heavy metals in dietary supplements should be established by regulatory authorities. Furthermore, it is required that manufacturers assess their products for compliance with standards. Environmental pollution has even reached the pristine places. Even in an isolated location such as Mount Everest heavy metals Pb, Cd, Cr, As, and Hg were detected. Yeo and Langley-Turnbaugh (2010) found that all snow samples from Everest had As and Cd levels higher than the USEPA drinking water guidelines. Besides, all soil samples were highly polluted with As (Yeo and Langley-Turnbaugh, 2010).

**TABLE 2:** Daily consumption limits for the five heavy metals (μg/day)^a^

	Hg	Pb	Cr	Cd	As
A	—	0.5	8.2	4.1	—
B	20	20	—	6	10
C	21 (inorganic)	—	210	70	21 (inorganic)
D	50	250	120	55	150
E	16	250	—	70	150
F	2	10	—	4.1	10
G	16 (methyl)	—	63 (hexavalent)	25	210

A, The California Safe Drinking Water and Toxic Enforcement Act (California Proposition 65) maximum allowable dose levels for chemicals causing reproductive toxicity, μg/day; B, American National Standards Institute (ANSI)/International Dietary Supplement Standard 173 = ANSI 173 Guidelines to the Dietary Supplement; C, United States Environmental Protection Agency (EPA) daily reference doses, μg/day; D, The Food and Agricultural Organization/World Health Organization Joint Expert Committee on Food Additives Acceptable Daily Intakes; E, Europe/WHO; F, American Herbal Products Association (AHPA); G, The European Food Safety Authority (EFSA).

^a^ A few regulation presented limits as tolerable weekly intake. Such thresholds were considered and shown as the maximum daily exposure in Table 2.

### Heavy Metals Binding to Thiol Group

**FIGURE 3:**
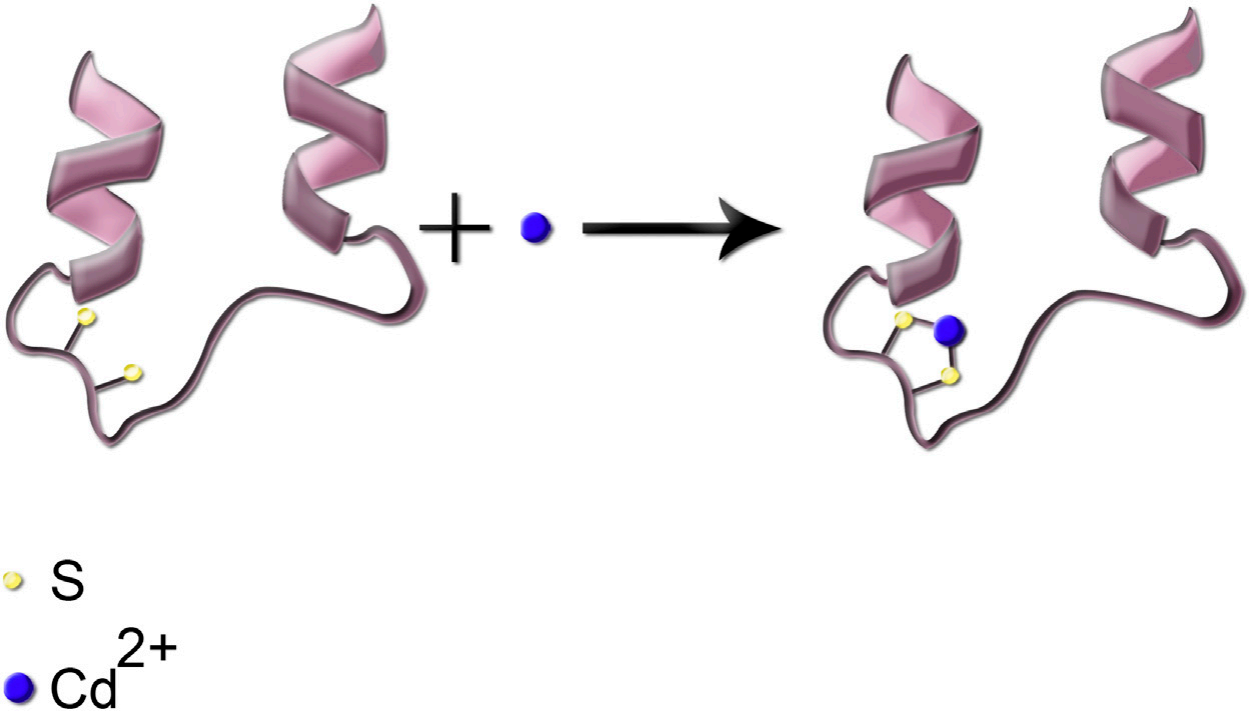
Simplified schematic presentation of the reaction of Cd metal ion with the thiol functional group; the two sulfhydryl groups are shown in cream color.

Following the entrance into the body, heavy metals can bind to lipids, proteins, and nucleic acids. Binding to enzymes and proteins commonly occurs through thiol (–SH) groups and modifies cysteine residues in proteins. As an example, reaction of Cd with the sulfhydryl group (–SH) in a protein is shown in Figure 3. Such protein inactivation is capable of disrupting the intracellular redox state. Consequently, an imbalanced antioxidant defense contributes to the development of liver injury. Similar reactions can occur with the other heavy metals. In complexation of heavy metals with thiol-containing proteins, the ligands are amino acids which contain the –SH functional group (Burford et al., 2005).

### ROS Generation

**FIGURE 4:**
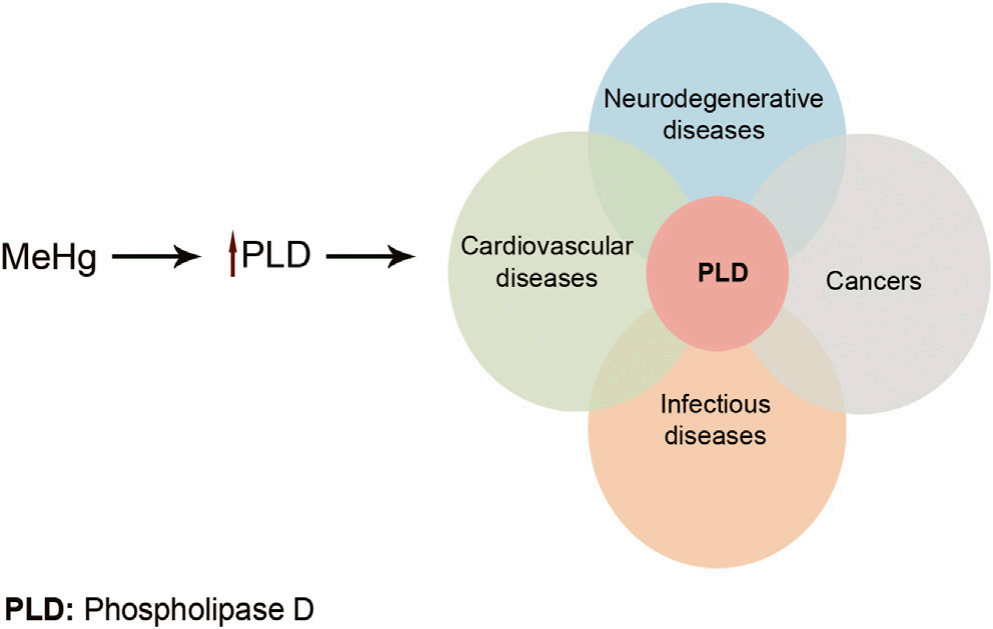
Me-Hg induces PLD activation. Increased PLD activity can lead to many diseases and cancers. Adapted from Brown et al. (2017) .

Toxic metals are able to generate free radicals, mainly ROS and RNS, which can induce oxidative stress. For example, arsenic has been shown to produce superoxide (O_2_^−**.**^), oxygen (O_2_^**.**^), nitric oxide (NO^**.**^), hydrogen peroxide (H_2_O_2_), and peroxyl (ROO^**.**^) radicals. Pb exposure significantly reduces antioxidant parameters GPx, CAT, SOD, GST, and GSH while it increases oxidative parameters MDA and H_2_O_2_ (Wang et al., 2013). ROS and RNS generation induced by Cr (VI) depletes cellular antioxidant power which results in oxidative stress and consequently the toxicity of DNA, lipid, and proteins (Yao et al., 2008; Aggarwal et al., 2019). Cd could indirectly generate O_2_^−**.**^, hydroxyl (OH), and NO^**.**^ radicals which could overwhelm cells’ antioxidant defense (Rani et al., 2014). It is believed that the toxic effects of Hg on the CNS and cardiovascular system are related to the increase in ROS production. This causes a reduction in the activity of antioxidant enzymes: glutathione peroxidase, catalase, and SOD. Moreover, high affinity of Hg for –SH groups can lead to decreased glutathione peroxidase activity and also interference with intracellular signaling of many receptors. Furthermore, Me-Hg has the capacity to induce phospholipase D (PLD) activation. Increased PLD activity has been shown to be involved in many human cancers and diseases (Figure 4) (Fernandes Azevedo et al., 2012; Brown et al., 2017). Pb and Hg toxicity is mediated either directly via ROS production or indirectly through the depletion of cellular antioxidants. However, Cd is believed to generate ROS indirectly. This may be due to the replacement of iron and copper by Cd in cellular proteins. The oxidative stress is then as a result of excess Fe and Cu accumulation. Moreover, replacement of the essential minerals disrupts the cell's biological metabolism. Alternatively, Cd could disrupt the antioxidant glutathione which will eventually lead to oxidative stress (Liu et al., 2009; Wu et al., 2016).

### The Carcinogenicity of Heavy Metals

The mechanism of carcinogenicity of heavy metals is unclear and complicated. Carcinogenicity of some heavy metals is assumed to be due to their bonding to regulatory proteins that are involved in cell cycle regulation, DNA synthesis and repair, and apoptosis (Kim et al., 2015). Besides, toxicogenomic investigations highlight the characterization of gene expressions after metal toxicity. Transcription factors such as Activator Protein 1 (AP-1), Nuclear Factor-kappa B (NF-κB), and p53 are targets for Cd and As. As a result, controlling the expression of protective genes failed, leading to uncontrolled cell growth and division (Valko et al., 2005). A few studies have examined Ras proteins mutations or increased activation in carcinogenic heavy metals exposure. Ngalame et al. (2014) showed overexpression of Ras in human prostate epithelial cells following As exposure. In another study, the elevation of the level of ERK 1/2, as well as transcription factors jun and fos, was observed *in vitro* by Cd. Cr (VI) also induced overexpression of jun in cultured cells. The mutated Ras protein loses its ability to be inactivated and the kinase cascade is not turned off. Besides, intensified jun and fos or activated ERK 1/2 continues repeatedly the gene expression. Thus, a permanently activated signaling pathway results in continuously activated proliferation, leading to increased tumor formation (Zuo et al., 2012; Ngalame et al., 2014; Ataei et al., 2019).

Arsenic is shown to inhibit DNA repair by inhibiting poly ADP-ribose polymerase 1 (PARP-1), a responsible enzyme for DNA break repair processes (Ding et al., 2009). Resistance to apoptosis due to heavy metals exposure breaks down a basic cell defense. It has been shown that Cd can induce malignant transformation of the prostate epithelial cell line through increased apoptotic resistance. Apoptotic resistance is attributed to the overexpression of Bcl-2 and disruption of a specific pathway of apoptosis known as the JNK pathway (Qu et al., 2007). Altered expression of genes involved in apoptosis and DNA repair is proposed for resistance to apoptosis induced by Cr^6+^ (Pritchard et al., 2005). Moreover, hexavalent Cr causes DNA damage by inactivation of DNA ligase, DNA polymerase β, and PARP-1. Deficient DNA repair due to Cr leads to chromosome instability which is a key mechanism of Cr^6+^-induced carcinogenesis (Wise et al., 2018).

### Epigenetic Mechanisms of Heavy Metals

Heavy metals can promote epigenetic alterations, including DNA methylation and histone modification. Hg, Pb, Cr, Cd, and As are capable of DNA methylation. Besides, Hg, Pb, Cr, and As can make histone alterations. However, there are no data available for Cd to induce histone modifications. On the other hand, only Cd and As are reported to mediate expression of noncoding RNAs, another molecular mechanism of epigenetic regulation. The exact mechanism of epigenetic changes due to heavy metals exposure is not fully determined. It seems that increased expression of protooncogenes and silencing of tumor suppresser genes via intracellular ROS production are the underlying causes (Cheng et al., 2012). DNA methylation is well known to inhibit the expression of some tumor suppressors. As a result, changes in gene expression result in the alteration of the cellular division process and facilitation of malignant transformation of cells (Li and Chen, 2016). Epigenetic dysregulations in human bronchial epithelial cells were reported following Cr^6+^ exposure. Wang Z. et al. (2018) found increased levels of histone H3 methylation marks (H3K9me2 and H3K27me3) and histone methyltransferases (HMTases) (Wang Z. et al., 2018). Hu et al. reported Cr-induced methylation of the p16 gene, a tumor suppressor gene, in the same cell line (Hu et al., 2016). Similarly, epigenetic changes may attribute to the Cd and As toxic and carcinogenic effects. DNA methylation as well as specific histone modification marks are associated with exposure to Cd and As (Bailey et al., 2013; Sanders et al., 2014; Xiao et al., 2015; Ma et al., 2016).

### Comparison of the Mechanistic Action

Since there are multiple cellular targets, the mechanism underlying the toxicity of toxic heavy metals is complicated. It is understood that Hg, Pb, Cr, Cd, and As commonly exert their toxicity through similar pathways including ROS generation, weakening of the antioxidant defense, enzyme inactivation, and multiple organ toxicity. For instance, glutathione peroxidase and reductase are known enzymes which are inactivated by these five metals. However, some metals selectively bind to a specific protein as a unique mechanism of that metal. For example, Pb-induced anemia occurs via the inhibition of two enzymes of heme biosynthesis: ALAD and ferrochelatase (Figure 1). The interaction of protein phosphatases (PP) with Cd can be mentioned as another example. Examination of protein phosphatases 1 (PP1) metal inhibitors showed that Cd^2+^ and Hg^2+^ both have inhibitory effects on PP1 enzyme. However, Cd^2+^ is likely a potent and competitive inhibitor of the enzyme (Pan et al., 2013). Heavy metals can disrupt endocrine systems by interfering mainly with steroid and thyroid hormones in human and animals. As an example, Cd, Pb, and Cr are known to induce thyrotoxicosis (Qureshi and Mahmood, 2010). The mechanism of toxicity of these heavy metals and the organ toxicity are presented in Table 1.

While different types of mercury compounds are found in the environment, the organic forms of Hg are more toxic than the inorganic species. Hg can cause cognitive impairment and CNS injury. Besides, Hg causes renal dysfunction especially in the proximal tubules due to its preferential accumulation in the kidneys. It has also harmful effects on liver and the intestine (Kim et al., 1995; Cheng et al., 2006).

The effect of proinflammatory cytokines in the brain following Pb exposure is inflammation in the CNS which in turn causes neurotoxicity (Struzynska et al., 2007). Pb has also shown to have hematotoxic and hepatotoxic effects. Moreover, it poses renal and respiratory harmful effects. Pb-induced renal toxicity might lead to necrosis. Apoptosis in lead toxicity takes place via the inhibition of Bcl-2 and the activation of caspase-3 following mitochondrial cytochrome C release (Liu et al., 2012). Lead neurotoxicity and cognitive impairment might be promoted via the inhibition of NMDA receptor and the blockage of neurotransmitter release (Neal and Guilarte, 2010).

Hexavalent chromium has been classified as a carcinogen. Cr toxicity can cause the lungs and upper respiratory cancers. Mechanisms of Cr toxicity and carcinogenicity are considered as DNA damage, genomic instability, and ROS generation. Following exposure and bioaccumulation of Cr, a variety of diseases appeared. Skin, hepatic, renal, reproductive, and neurological disorders can be mentioned (Fang et al., 2014). Moreover, many other cellular injuries and cell death as both apoptosis and necrosis may occur (De Flora et al., 2008).

Like Cr, Cd is also classified as a carcinogen. Consequences of Cd poisoning might be painful degenerative bone disease, kidney failure, and the GI and lungs diseases (Nishijo et al., 2017). Cd can bind to MT and other metal transporters in the small intestine and accumulates in the GI tract (Ohta and Ohba, 2020). Altered miRNA expression due to Cd exposure may play a role in Cd-induced nephrotoxicity (Fay et al., 2018). Apoptosis is associated with the cytochrome C release into the cytosol due to Cd exposure (Pham et al., 2006).

Like chromium and cadmium, arsenic is considered a human carcinogen. Chronic arsenic toxicity or arsenicosis causes skin manifestations such as pigmentation and keratosis. Arsenic poisoning may exert obstructive and restrictive pulmonary diseases. Arsenic has been linked to cognitive dysfunction and neurological problems such as pricking sensation in hands and legs (Lee et al., 2006; Wasserman et al., 2011).

## Conclusion

The heavy metals enter the body from different ways including drinking water, air, food, or occasionally dermal exposure. Following absorption, heavy metals are retained, and they accumulate in the human body. Bioaccumulation of toxic metals leads to a diversity of toxic effects on a variety of body tissues and organs. Metal toxicity can have acute or chronic manifestations. Heavy metals disrupt cellular events including growth, proliferation, differentiation, damage-repairing processes, and apoptosis. Toxic metals can also promote epigenetic alterations which can influence gene expression. Comparison of the mechanisms of action reveals similar pathways for these metals to induce toxicity including ROS generation, weakening of the antioxidant defense, enzyme inactivation, and oxidative stress. On the other hand, some researches have shown that the metals selectively bind to specific macromolecules. The interaction of Pb with ALAD and ferrochelatase is within this context. Reactions of other heavy metals with certain proteins were discussed as well. Some toxic metals including Cr, Cd, and As cause genomic instability. Defects in DNA repair following the induction of oxidative stress and DNA damage by these metals is considered as the cause of their carcinogenicity. The application of chelation therapy for the management of metal poisoning has not been reviewed here. This could be another aspect of heavy metals to be reviewed in the future. Developing specific biomarkers for monitoring heavy metals will be a major achievement in the field. Future research will benefit from the evaluation of new targets as protective procedures against organ toxicity induced by heavy metals.

## Glossary

BBB: blood brain barrier
GI: gastrointestinal
ALT: alanine transaminase
AST: aspartate transaminase
ALP: alkaline phosphatase
Oat3: organic anion transporter 3
ROS: reactive oxygen species
GGT: gamma glutamyl transferase
CNS: central nervous system
ET-1: endothelin-1
NO: nitric oxide
EPO: eosinophil peroxidase
OA: ovalbumin
WBC: white blood cells
RBC: red blood cells
AP: acid phosphatase
LDH: lactate dehydrogenase
GPx: glutathione peroxidase
CAT: catalase
SOD: superoxide dismutase
GST: glutathione S-transferase
GSH: glutathione
MDA: malondialdehyde
COX-2: cyclooxygenase-2
LPO: lipid peroxidation
4-HAD|: 4-hydroxyalkenals
ALAD: δ-aminolevulinic acid dehydratase
IFN-γ: interferon-γ
NK: natural killer
PHA: phytohemagglutinin
PBMC: peripheral blood mononuclear cell
PFT: pulmonary function tests
IgE: immunoglobulin E
BLL: blood lead level
SBP: systolic blood pressure
DBP: diastolic blood pressure
Hb: hemoglobin
Hct: hematocrit
MCV: mean corpuscle volume
MCH: mean corpuscle hemoglobin
IARC: International Agency for Research on Cancer
SIRs: standardized incidence ratios
NTP: National Toxicology Program
MT: metallothionein
DMT1: divalent metal transporter 1
ZIP14: zinc importing protein 14
TRPV6: Transient Receptor Potential Vanilloid 6
Ca: calcium
Drp 1: dynamin-related protein 1
NMDA: N-methyl-d-aspartate
PSD-95: postsynaptic density protein 95
pp-CaMKII: calcium/calmodulin-dependent protein kinase II
